# C-Reactive Protein to Albumin Ratio Predicts 30-Day and 1-Year Mortality in Postoperative Patients after Admission to the Intensive Care Unit

**DOI:** 10.3390/jcm7030039

**Published:** 2018-02-26

**Authors:** Tak Kyu Oh, Eunjeong Ji, Hyo-seok Na, Byunghun Min, Young-Tae Jeon, Sang-Hwan Do, In-Ae Song, Hee-Pyoung Park, Jung-Won Hwang

**Affiliations:** 1 Department of Anaesthesiology and Pain Medicine, Seoul National University Bundang Hospital, 82, Gumi-ro 173 Beon-gil, Bundang-gu, Seongnam-si, Gyeonggi-do 463-707, Korea; airohtak@hotmail.com (T.K.O.); hsknana@gmail.com (H-s.N.); 54309@snubh.org (B.M.); ytjeon@snubh.org (Y.-T.J.); 00782@snubh.org (S.-H.D.); songoficu@outlook.kr (I.-A.S.); 2 Medical Research Collaborating Center, Seoul National University Bundang Hospital, 82, Gumi-ro 173 Beon-gil, Bundang-gu, Seongnam-si, Gyeonggi-do 463-707, Korea; 99145@snubh.org; 3 Department of Anesthesiology and Pain Medicine, Seoul National University Hospital, Seoul National University College of Medicine, 101 Daehakro, Jongno-gu, Seoul 03080, Korea; hppark@snu.ac.kr

**Keywords:** critical care, intensive care units, sepsis, surgery

## Abstract

C-reactive protein and albumin are associated with poor outcomes in critically ill patients. This study aimed to investigate the significance of the C-reactive protein/albumin (CRP/ALB) ratio as a novel prognostic factor for 30-day or 1-year mortality after admission to the postoperative intensive care unit (ICU). This retrospective study was conducted by examining the medical records of postoperative patients aged 19 years or older admitted to the ICU at a tertiary care hospital from January 2007 to July 2016. This study included data on 11,832 postoperative patients in the analysis. The cut-off value of the CRP/ALB ratio was set at 1.75 and 1.58 for 30-day and 1-year mortality after ICU admission, respectively. In postoperative patients with a high CRP/ALB ratio (>1.75 or >1.58), the probability of death within 30 days or 1 year after ICU admission were 30% or 43% higher than that in the group with the least CRP/ALB ratio, respectively (≤1.75 or ≤1.58)(*p* < 0.05). This study found the CRP/ALB ratio measured immediately after ICU admission to be an independent risk factor for 30-day and 1-year mortality in postoperative patients admitted to the ICU.

## 1. Introduction

The C-reactive protein (CRP) and albumin are widely used as serum inflammatory markers to predict mortality in critically ill patients [1,2]. The prognostic roles of CRP and albumin can be explained by their abilities to reflect inflammation in the acute phase in critical settings [3] and in the malnutrition status of critically ill patients, respectively [4,5]. Hence, many studies have recently investigated whether the ratio of CRP to ALB can effectively reflect the prognosis of critically ill patients diagnosed with severe sepsis or septic shock [6,7]. These previous studies have reported that the CRP/ALB ratio measured on admission was an independent risk factor in patients with severe sepsis or septic shock.

However, few studies have ever attempted examining the prognostic value of the CRP/ALB ratio in all general ICU patients or in postoperative patients admitted to the ICU. Given that a surgical stress-induced inflammatory response occurs during the postoperative period [8], a number of studies have claimed that postoperative CRP/ALB ratio is an indicator of postoperative prognosis [9,10]. Therefore, the CRP/ALB ratio measured on admission to the ICU may be highly associated with the outcome in postoperative ICU patients.

The present study, therefore, aimed to investigate whether CRP/ALB ratio on admission to the ICU in postoperative patients is associated with the 30-day and 1-year mortality after ICU admission. It also aimed to determine the optimal cut-off point of CRP/ALB ratio in postoperative patients on ICU admission and to examine this as a novel prognostic factor in this study.

## 2. Materials and Methods

This retrospective observational study obtained approval from the Institutional Review Board of Seoul National University Bundang Hospital (SNUBH) (approval number: B-1710/424-104; approval date: 26 September 2017). The SNUBH is a tertiary care hospital with 1360 beds, of which 102 were critical care beds (medical, surgical, neurologic, and emergency I and II). Additionally, nearly 150 elective or emergency operations were performed in 38 operating rooms. The institution has been maintaining accurate medical records after establishing an electronic medical record system in 2003. This study examined the medical records of patients aged 19 years or older who underwent either elective or emergency surgery at SNUBH and were admitted to the ICU from 1 January 2007 to 31 July 31 2016. During the study period, the indication for ICU admissions in postoperative patients was determined by the anesthesiologists based on the underlying disease and surgical severity. For those who were admitted to the ICU more than twice, only the last admission was included in this study. We excluded those who did not undergo laboratory tests to provide CRP and albumin data on the same day after the postoperative ICU admission. Additionally, postoperative ICU admissions after major hepatectomy or liver transplantation were excluded from the analysis. This is because severe liver dysfunction could cause the deficiency of CRP or albumin production.

At baseline, we collected demographic data, surgery-related data, history of underlying diseases, laboratory data after ICU admission, and accurate death dates of postoperative patients. With a history of underlying hypertension and diabetes mellitus, pre-admission diagnosis and regular intake of medication were added to the inclusion criteria. The American Society of Anesthesiologists classification was based on the patient’s immediate records prior to surgery. The Acute Physiology and Chronic Health Evaluation II (APACHE II) score was based on the patient’s records on ICU admission. Preoperative laboratory results were tested within one month before surgery. However, the CRP or albumin data used in this study were the results of laboratory tests performed, using venous or arterial blood samples of postoperative patients within 24 h of ICU admission. If multiple tests were performed, the results of the test closest to the time of postoperative ICU admission were used for the analysis. During the study period, CRP (mg L^–1^) and albumin (g L^–1^) were measured consistently by the Department of Laboratory Medicine (normal ranges: 0–10 of CRP in mg L^–1^, and 35–50 of albumin in g L^–1^).

Under the approval of the Ministry of Interior and Safety in the Republic of Korea, the exact date of death was set as 14 August 2017 for all patients including those who were lost to follow-up.

In this study, we investigated whether the CRP/ALB ratio, a primary outcome indicator, has effect on 30-day and 1-year mortality in postoperative patients after ICU admission using the cut-off point of the CRP/ALB ratio set for that purpose.

The baseline characteristics of all patients were presented as median with interquartile range or number with percent. We set the optimal cut-off point for the CRP/ALB ratio by using the receiver operating characteristics (ROC) curve analysis for 30-day and 1-year mortality after postoperative ICU admission. Furthermore, a univariate Cox regression analysis was performed to determine the probability of 30-day or 1-year death in postoperative patients after ICU admission. We fitted a multivariate Cox regression model with significant variables (*p* < 0.05) from the univariate Cox regression analysis. To prevent interactions among variables, the APACHE II score, CRP, and duration in the hospital were excluded. To verify whether each variable in the selected multivariate Cox regression model satisfied the Cox proportional hazard assumptions, the ‘log minus log plot’ was used. Among the variables, ‘emergency operation’ and ‘diagnosis of cancer’ failed to satisfy the assumptions and were included in the Cox proportional hazard model as stratification variables. The analyses were performed using the IBM SPSS 24.0 and R version 3.3.2. (IBM SPSS Statistics for Windows, IBM Corp Armonk, NY, USA). A *p* value of < 0.05 was considered statistically significant.

## 3. Results

**Figure 1:**
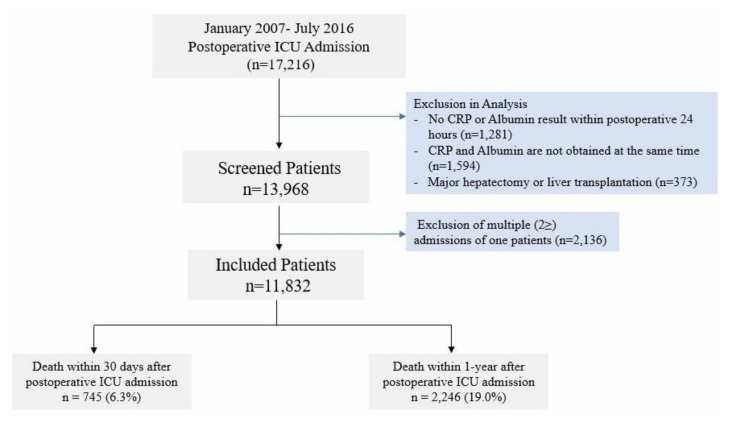
Patients’ flow chart. CRP: C-reactive protein; ALB: albumin.

A total of 17,216 postoperative patients were admitted to the ICU at SNUBH from 1 January 2007 to 31 July 2016. Of these patients, 1281 cases were excluded because a serum CRP test or serum albumin test was not performed on the same admission day to the ICU. A total of 1594 cases where the CRP and albumin levels were not simultaneously obtained from the venous or arterial sample were also excluded from the analysis. Additionally, 2136 cases were excluded because the corresponding patients were admitted to the ICU more than twice. Lastly, 373 cases of postoperative admissions after major hepatectomy or liver transplantation were excluded from the analysis. Finally, data on 11,832 cases were analyzed (Figure 1). The baseline characteristics of all patients are presented in Table 1. Overall, 745 (6.3%) and 2246 (19.0%) of postoperative patients died within 30 days and 1 year, respectively.

### 3.1. Cut-off Point of CRP/ALB Ratio for Postoperative 30-Day and 1-Year Mortality after ICU Admission

**Figure 2:**
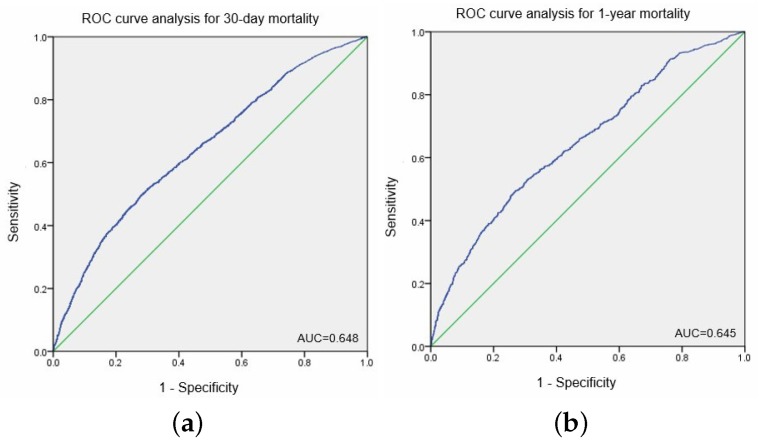
Receiver operative curve analysis for 30-day mortality (**a**) and 1-year mortality (**b**) after postoperative ICU admission. Area under the curve for 30-day mortality: 0.648. Area under the curve for 1-year mortality: 0.645. CRP; C-reactive protein; ALB, Albumin; ICU, Intensive Care Unit.

Based on ROC curve analysis for postoperative 30-day and 1-year mortality after ICU admission, area under the curve was 0.648 and 0.645, respectively (Figure 2a,b). The optimal cut-off point of CRP/ALB ratio, with equal values of sensitivity and specificity, were 1.75 for postoperative 30-day mortality and 1.58 for 1-year mortality after ICU admission.

### 3.2. Cox Proportional Hazard Model in Relation to 30-Day and 1-Year Mortality in Postoperative Patients after Admission to the ICU

Table 2 shows the results of univariate and multivariate Cox proportional hazard models for the analysis of 30-day mortality in postoperative patients after ICU admission. The Cox proportional hazard model revealed that an increase of 1 for the CRP/ALB ratio was associated with an 7% increase in 30-day mortality risk (hazard ratio (HR): 1.07; 95% confidence interval (CI): 1.05–1.09; *p* < 0.001). Furthermore, the probability of death following ICU admission in the high CRP/ALB group (>1.75) was 30% higher than that in the low CRP/ALB group (≤1.75) (HR: 1.30; 95% CI: 1.11–1.53; *p* < 0.001).

Table 3 shows the results of the univariate and multivariate Cox proportional hazard models for the analysis of 1-year mortality in postoperative patients after ICU admission. The Cox proportional hazard model revealed that an increase of 1 for the CRP/ALB ratio was associated with an 8% increase in 30-day mortality risk (HR: 1.07; 95% CI: 1.06–1.09; *p* < 0.001). Furthermore, the probability of death following ICU admission in the high CRP/ALB group (>1.58) was 43% higher than that in the low CRP/ALB group (≤1.75) (HR: 1.43; 95% CI: 1.31–1.57, *p* < 0.001).

### 3.3. Subgroup Analysis According to Type of Surgery, Diagnosis of Cancer, and ICU Admission after Emergency Surgery for 1-Year and 30-Day Mortality

Table 4 shows the results of the subgroup analysis in relation to postoperative 30-day and 1-year mortality after ICU admission according to types of surgery, diagnosis of cancer, and ICU admission after emergency surgery. Orthopedic, obstetric, and gynecologic surgery, and the urologic surgical group showed the highest postoperative 30-day mortality among types of surgeries according to CRP/ALB ratio (HR: 1.17; 95% CI: 1.10–1.24; *p* < 0.001). In addition, postoperative 1-year mortality after ICU admission according to CRP/ALB ratio was highest in the cardiovascular or thoracic surgical group (HR: 1.11; 95% CI: 1.08–1.13; *p* < 0.001). Diagnosis of cancer before surgery was associated with increasing postoperative 30-day and 1-year mortality after ICU admission (*p* < 0.001), while ICU admission after emergency surgery was associated with increasing postoperative 1-year mortality (*p* = 0.006) only.

## 4. Discussion

The present study found that 30-day and 1-year mortality following postoperative admission to the ICU varied independently with the CRP/ALB ratio on admission, with a positive correlation. This significant finding was obtained in the multivariate Cox proportional hazard model which reflected the types of surgery performed, status of underlying disease, and various laboratory data available on ICU admission. We also suggested the cut-off point of 1.58 and 1.75 that was independently associated with 30-day and 1-year mortality, respectively in postoperative patients after ICU admission. The values can be used as a standard in clinical practices.

The most important issue in this study was the process of determining the cut-off point of the CRP/ALB ratio. In the present study, we derived the cut-off point using the ROC curve analysis, as 0.648 for 30-day mortality and 0.645 for 1-year mortality after ICU admission. The resulting value was 1.75 for 30-day mortality and 1.58 for 1-year mortality after ICU admission, with equal values of sensitivity and specificity. Many previous studies regarding CRP/ALB ratio used ROC curve analysis to determine the cut-off values affecting prognosis of the patients [6,7,11]. The cut-off values differed between studies, thus reflecting the differences in the characteristics of the patients in these studies. It is, therefore, necessary to set the reference value of the CRP/ALB ratio by taking into account the characteristics of the study population. For examples, patients with sepsis or septic shock had higher value of CRP/ALB ratio as a cut-off value than in our study. Ranzani et al. [7] proposed the CRP/ALB ratio cut-off point as 8.7 on ICU admission and 2.0 at discharge. Kim et al. [6] subsequently suggested the cut-off value of 5.09 on ICU admission and 0.91 at 74 hours after ICU admission. Considering that the patients with sepsis usually had higher values of inflammatory markers such as CRP or procalcitonin [12], CRP/ALB ratio in these patients would have higher ratio than with other studies including our study.

Secondly, CRP/ALB ratio for cancer patients should also be considered. The CRP/ALB ratio was initially used as a novel prognostic factor in patients with cancer [13]. However, previously, in 10 studies, the cut-off point of 0.0300-0.6712 was used when examining the association between the CRP/ALB ratio with cancer outcomes in patients with solid cancers [11]. Many postoperative patients in our study were cancer patients (3875, 32.8%); and being diagnosed of cancer was significantly associated with increased 30-day and 1-year mortality after ICU admission in our subgroup analysis. Third, the postoperative setting characteristics should also be considered. The present study can be differentiated from other studies based on the fact that it used the CRP/ALB ratio measured immediately after postoperative ICU admission. The CRP level reflects a complex interaction of stressful conditions such as surgical invasiveness and infection sign [8]. On the other hand, the albumin level is directly influenced by the patient’s underlying nutritional status and the perioperative care including fluid loss [14,15]. Thus, it is better to combine CRP and albumin to predict the postoperative outcome. Such a combination would be definitely required for critically ill patients who require postoperative ICU care. In the present study, we made a novel attempt using the CRP/ALB ratio measured immediately after postoperative ICU admission in adult patients rather than a limited population of patients specific to the types of disease or surgery and thereby, proved the prognostic significance of the CRP/ALB ratio.

Lastly, the relationship between CRP/ALB ratio and liver dysfunction could be an important issue for prognosis in postoperative ICU patients in our study. Progression of chronic liver disease or hepatic dysfunction had a positive correlation with CRP and negative correlation with albumin [16]. Furthermore, the resection of the liver could be a significant risk factor for postoperative hepatic dysfunction [17], and it might have elevated CRP/ALB ratio in the postoperative period. We excluded major hepatectomy or liver transplantation from our analysis to avoid the impact of surgery-induced hepatic impairment. However, there were 2013 (17.0%) patients with preoperatively increased liver enzymes (aspartate aminotransferase, or alanine aminotransferase > 40 U L^–1^), which could affect our outcome regarding CRP/ALB ratio. However, all preoperative laboratory tests including liver enzymes were included in our multivariate Cox proportional hazard model for the 30-day or 1-year mortality. Therefore, non-surgery-induced preoperative hepatic dysfunction was considered to have had an effect on the CRP/ALB ratio in our study among the patients.

This study had several limitations. First, as commonly found in studies conducted at a retrospective single center, bias may exist in the results, and the lack of generalizability cannot be ruled out. Second, the study population might be considered heterogeneous in the absence of quantifiable and specific postoperative ICU admission indications. Third, the CRP and albumin tests were not performed simultaneously on all patients, although we included those who had the CRP and albumin data measured within 24 h after admission to the ICU. Fourth, the exact death date was confirmed for all the patients; however, we could not identify the specific causes of death. Finally, analysis was not conducted on the training cohort and the validation cohort separately. However, since the general cut-off of CRP/ALB ratio had not been determined previously, we concluded that it would be better to present a cut-off for the cohort with many patients. Therefore, this study focused on the suggestion of a cut-off for the cohort in our hospital as the training cohort.

## 5. Conclusions

In conclusion, the present study identified the CRP/ALB ratio measured immediately after admission to the ICU as an independent risk factor for 30-day and 1-year mortality in postoperative patients receiving ICU care.

## Figures and Tables

**Table 1:** Baseline characteristics of patients.

Total (*n* = 11,832)	Number (Percent)	Median (IQR)
Gender, male	6979 (59.0%)	-
Age (year)	-	66 (53–74)
Body mass index (kg m^–2^)	-	23.3 (20.9–25.7)
Operative characteristics		
- Operation time (h)	-	3.4 (1.7–5.0)
- Emergency operation	1290 (10.9%)	-
- Surgery type		
- *Cardiovascular or thoracic surgery*	4642 (39.2%)	-
- *General surgery*	2663 (22.5%)	-
- *Neuro or spine surgery*	2134 (18.0%)	-
- *Neuro or spine surgery*	1873 (15.8%)	-
- *Orthopedic, OBGY, urologic surgery*	520 (4.4%)	-
Duration in ICU (h)	-	23.0 (18.0–51.0)
Duration in hospital (day)	-	16.0 (11.0–30.0)
Preoperative morbidity		
- APACHE II	-	22.0 (15.0–28.0)
- ASA classification		
- *I*	1372 (11.6%)	-
- *II*	4723 (39.9%)	-
- *III*	4863 (41.1%)	-
- *IV + V + VI*	874 (7.4%)	-
- History of hypertension	2588 (21.9%)	-
- History of ischemic heart disease	1891 (16.0%)	-
- History of diabetes mellitus	1227 (10.4%)	-
- Diagnosis of cancer	3875 (32.8%)	-
Preoperative laboratory test results within one month		
- Blood urea nitrogen (mg dL^–1^)	-	14 (11–20)
- Creatinine (mg dL^–1^)	-	0.8 (0.6–1.1)
- Aspartate aminotransferase (IU L^–1^)	-	26 (19–44)
- Alanine aminotransferase (IU L^–1^)	-	18 (11–30)
- Hemoglobin (g dL^–1^)	-	11.2 (9.9–12.7)
- Platelet (10^3^ μL^–1^)	-	183 (133–242)
- White blood cell (10^3^ μL^–1^)	-	10.4 (7.5–14.1)
- Serum sodium (mmol L^–1^)	-	139 (136–141)
- Serum potassium (mmol L^–1^)	-	4.0 (3.7–4.3)
Laboratory results within 24 h after ICU admission time		
- C-reactive protein (mg L^–1^)	-	40.2 (6.0–88.4)
- Albumin (g L^–1^)	-	32 (27–37)
- C-reactive protein/albumin ratio	-	1.2 (0.1–4.8)
1-year mortality after ICU admission	-	2246 (19.0%)
30-day mortality after ICU admission	-	745 (6.3%)

Values are expressed as the median (IQR) or number (percentage). IQR, interquartile; CRP, C-reactive protein; APACHE, Acute Physiology and Chronic Health Evaluation; ICU, intensive care unit; OBGY, obstetric and gynecologic; OPH, ophthalmic; ENT, ear-nose-throat; DT, dental; ASA, American Society of Anesthesiologists.

**Table 2:** Univariate and multivariate Cox regression analysis in relation to 30-day mortality after postoperative ICU admission.

Variable	Univariate Analysis		Multivariate Analysis	
	Hazard Ratio (95% CI)	*p*-Value	Hazard Ratio (95% CI)	*p*-Value
Gender: male (vs. female)	1.05 (0.90–1.21)	0.556	-	-
Age	1.02 (1.02–1.03)	<0.001	1.02 (1.01–1.02)	<0.001
Body mass index (kg m^–2^)	0.94 (0.92–0.95)	<0.001	0.97 (0.95–0.98)	<0.001
- <18.5 vs. 18.5–25.0	1.61 (1.30–2.00)	<0.001	1.29 (1.03–1.61)	0.024
- >25 vs. 18.5–25.0	0.72 (0.60–0.86)	<0.001	0.84 (0.70–1.00)	0.050
Operation time (h)	0.96 (0.93–1.00)	0.007	0.96 (0.93–0.98)	0.003
Emergency operation	1.86 (1.55–2.25)	<0.001	1.32 (1.08–1.62)	0.007
APACHE II	1.10 (1.09–1.11)	<0.001	-	-
ASA classification				
- *I*	1	-	1	-
- *II*	0.87 (0.66–1.16)	0.345	0.86 (0.64–1.15)	0.309
- *III*	1.58 (1.21–2.06)	0.001	1.12 (0.85–1.47)	0.439
- *IV + V + VI*	3.50 (2.60–4.71)	<0.001	2.03 (1.50–2.76)	<0.001
Blood urea nitrogen (mg dL^–1^) (>20 vs. ≤20)	3.66 (3.17–4.22)	<0.001	1.55 (1.29–1.87)	<0.001
CRP (mg L^–1^) (>10 vs. ≤10)	2.19 (1.80–2.66)	<0.001	-	-
Creatinine (mg dL^–1^) (>1.3 vs. ≤1.3)	3.94 (3.40–4.56)	<0.001	1.72 (1.43–2.08)	<0.001
AST (IU L^–1^) (>40 vs. ≤40)	2.59 (2.25–3.00)	<0.001	1.56 (1.30–1.88)	<0.001
ALT (IU L^–1^) (>40 vs. ≤40)	2.25 (1.93–2.63)	<0.001	1.38 (1.13–1.68)	0.001
Hemoglobin (g dL^–1^) (<7 vs. ≥7)	4.62 (3.28–6.52)	<0.001	1.96 (1.33–2.89)	0.001
Platelet count (10^3^ uL^–1^) (<100 vs. ≥100)	3.15 (2.69–3.69)	<0.001	2.10 (1.76–2.49)	<0.001
White blood cell (10^3^ uL^–1^)				
- <4 vs. 4–10	2.93 (2.23–3.85)	<0.001	1.80 (1.35–2.41)	<0.001
- >10 vs. 4–10	1.21 (1.04–1.41)	0.016	1.13 (0.96–1.33)	0.132
Serum sodium (mmol L^–1^)				
- <135 vs. 135–145	2.58 (2.18–3.05)	<0.001	1.67 (1.39–2.00)	<0.001
- >145 vs. 135–145	4.55 (3.69–5.61)	<0.001	2.54 (2.02–3.19)	<0.001
Serum potassium (mEq L^–1^)				
- <3.5 vs. 3.5–5	1.80 (1.49–2.18)	<0.001	1.33 (1.09–1.63)	0.005
- >5 vs. 3.5–5	4.34 (3.44–5.47)	<0.001	2.22 (1.73–2.85)	<0.001
History of hypertension	1.54 (1.26–1.87)	<0.001	1.15 (0.90–1.46)	0.258
History of diabetes mellitus	1.47 (1.12–1.93)	0.006	1.24 (0.90–1.70)	0.184
History of ischemic heart disease	1.58 (1.25–1.99)	<0.001	1.57 (1.20–2.04)	0.001
Diagnosis of cancer	1.00 (0.86–1.17)	0.972	-	-
CRP/albumin (dichotomized)				
- ≤1.75	1	-	1	-
- >1.75	2.14 (1.85–2.47)	<0.001	1.30 (1.11–1.53)	0.001
CRP/albumin (continuous) *	1.08 (1.01–1.08)	<0.001	1.07 (1.05–1.09)	<0.001

HR of CRP/albumin (continuous) * was a value from another Cox proportional Hazard model without CRP/albumin (dichotomized). ICU, intensive care unit; BMI, body mass index; APACHE, Acute Physiology and Chronic Health Evaluation; OBGY, obstetric and gynecologic; OPH, ophthalmic; ENT, ear-nose-throat; DT, dental; ASA, American Society of Anesthesiologists; CRP, C-reactive protein; AST, aspartate aminotransferase; ALT, alanine aminotransferase.

**Table 3:** Univariate and multivariate Cox regression analysis in relation to 1-year mortality after postoperative ICU admission.

Variable	Univariate Analysis		Multivariate Analysis	
	Hazard Ratio (95% CI)	*p*-Value	Hazard Ratio (95% CI)	*p*-Value
Gender: male (vs. female)	1.23 (1.13–1.34)	<0.001	1.07 (0.98–1.15)	0.153
Age	1.03 (1.02–1.03)	<0.001	1.02 (1.02–1.03)	<0.001
Body mass index (kg m^–2^)				
- <18.5 vs. 18.5–25.0	2.10 (1.87–2.35)	<0.001	1.74 (1.54–1.95)	<0.001
- >25 vs. 18.5–25.0	0.60 (0.54–0.67)	<0.001	0.71 (0.64–0.79)	<0.001
Operation time (h)	0.99 (0.97–1.01)	0.232	-	-
Emergency operation	1.33 (1.18–1.51)	<0.001	1.16 (1.13–1.21)	0.009
APACHE II	1.05 (1.04–1.06)	<0.001	-	-
ASA classification				
- *I*	1	-	1	-
- *II*	1.08 (0.93–1.27)	0.324	0.94 (0.80–1.10)	0.437
- *III*	1.58 (1.36–1.84)	<0.001	1.26 (1.07–1.47)	0.005
- *IV + V + VI*	2.50 (2.08–3.00)	<0.001	1.86 (1.54–2.25)	<0.001
Blood urea nitrogen (mg dL^–1^) (>20 vs. ≤20)	2.75 (2.53–2.99)	<0.001	1.62 (1.45–1.79)	<0.001
CRP (mg L^–1^) (>10 vs. ≤10)	2.00 (1.80–2.23)	<0.001	-	-
Creatinine (mg dL^–1^) (>1.3 vs. ≤1.3)	2.57 (2.35–2.81)	<0.001	1.34 (1.19–1.49)	<0.001
AST (IU L^–1^) (>40 vs. ≤40)	1.57 (1.44–1.71)	<0.001	1.18 (1.05–1.31)	0.005
ALT (IU L^–1^) (>40 vs. ≤40)	1.58 (1.43–1.74)	<0.001	1.17 (1.04–1.33)	0.012
Hemoglobin (g dL^–1^) (<7 vs. ≥7)	2.74 (2.11–3.57)	<0.001	1.56 (1.17–2.07)	0.002
Platelet count (10^3^ uL^–1^) (<100 vs. ≥100)	1.87 (1.68–2.08)	<0.001	1.53 (1.37–1.71)	<0.001
White blood cell (10^3^ uL^–1^)				
- <*4 vs. 4–10*	2.27 (1.90–2.70)	<0.001	1.42 (1.18–1.71)	<0.001
- >*10 vs. 4–10*	1.13 (1.04–1.23)	0.006	1.07 (0.98–1.17)	0.120
Serum sodium (mmol L^–1^)				
- <135 vs. 135–145	2.43 (2.20–2.67)	<0.001	1.68 (1.52–1.86)	<0.001
- >145 vs. 135–145	2.37 (2.03–2.77)	<0.001	1.90 (1.62–2.24)	<0.001
Serum potassium (mEq L^–1^)				
- <3.5 vs. 3.5–5	1.52 (1.36–1.71)	<0.001	1.37 (1.21–1.54)	<0.001
- >5 vs. 3.5–5	2.91 (2.47–3.41)	<0.001	1.63 (1.37–1.93)	<0.001
History of hypertension	1.39 (1.25–1.56)	<0.001	1.24 (1.10–1.40)	<0.001
History of diabetes mellitus	1.10 (0.95–1.26)	0.194	-	-
History of ischemic heart disease	1.56 (1.37–1.78)	<0.001	1.38 (1.19–1.60)	<0.001
Diagnosis of cancer	1.43 (1.37–1.49)	<0.001	1.47 (1.41–1.54)	<0.001
CRP/albumin (dichotomized)				
- ≤1.58	1	-	1	-
- >1.58	2.06 (1.90–2.25)	<0.001	1.49 (1.36–1.62)	<0.001
CRP/albumin (continuous) *	1.08 (1.08–1.09)	<0.001	1.08 (1.07–1.09)	<0.001

HR of CRP/albumin (continuous) * was a value from another Cox proportional Hazard model without CRP/albumin (dichotomized). ICU, intensive care unit; BMI, body mass index; APACHE, Acute Physiology and Chronic Health Evaluation; OBGY, obstetric and gynecologic; OPH, ophthalmic; ENT, ear-nose-throat; DT, dental; ASA, American Society of Anesthesiologists; CRP, C-reactive protein; AST, aspartate aminotransferase; ALT, alanine aminotransferase.

**Table 4:** Subgroup analysis according to type of surgery, diagnosis of cancer, and ICU admission after emergency surgery for 1-year and 30-day mortality.

	CRP/Albumin for 30-Day Mortality		CRP/Albumin for 1-Year Mortality	
	Hazard Ratio (95% CI)	*p*-Value *	Hazard Ratio (95% CI)	*p*-Value *
Cardiovascular or thoracic surgery (*n* = 4642)	1.07 (1.03–1.12)	<0.001	1.11 (1.08–1.13)	<0.001
General surgery (*n* = 2663)	1.02 (0.98–1.08)	0.336	1.07 (1.05–1.10)	<0.001
Neuro or spine surgery (*n* = 2134)	1.08 (0.99–1.18)	0.082	1.09 (1.03–1.16)	0.003
Orthopedic, OBGY, urologic surgery (*n* = 1873)	1.17 (1.10–1.24)	<0.001	1.10 (1.06–1.14)	<0.001
Plastic, OPH, ENT, DT, procedures (*n* = 520)	1.11 (1.04–1.18)	0.001	1.05 (1.00–1.09)	0.036
Diagnosis of cancer (*n* = 3875)	1.10 (1.06–1.14)	<0.001	1.08 (1.06–1.10)	<0.001
ICU admission after emergency surgery (*n* = 1290)	1.02 (0.96–1.09)	0.474	1.05 (1.02–1.10)	0.006

* Hazard ratios were derived from multivariate Cox regression models using all covariates in 12 subgroup analysis. OBGY, obstetric and gynecologic; OPH, ophthalmic; ENT, ear-nose-throat; DT, dental; CRP, C-reactive protein; ICU, intensive care unit.

## References

[1] Devran O., Karakurt Z., Adiguzel N., Gungor G., Mocin O.Y., Balci M.K., Celik E., Salturk C., Takir H.B., Kargin F. (2012). C-reactive protein as a predictor of mortality in patients affected with severe sepsis in intensive care unit. Multidiscip. Respir. Med..

[2] Quispe E.A., Li X.M., Yi H. (2016). Comparison and relationship of thyroid hormones, il-6, il-10 and albumin as mortality predictors in case-mix critically ill patients. Cytokine.

[3] Povoa P. (2002). C-reactive protein: A valuable marker of sepsis. Intensive Care Med..

[4] Carriere I., Dupuy A.M., Lacroux A., Cristol J.P., Delcourt C. (2008). Pathologies Oculaires Liees a l’Age Study Group. Biomarkers of inflammation and malnutrition associated with early death in healthy elderly people. J. Am. Geriatr. Soc..

[5] Dominguez de Villota E., Mosquera J.M., Rubio J.J., Galdos P., Diez Balda V., de la Serna J.L., Tomas M.I. (1980). Association of a low serum albumin with infection and increased mortality in critically ill patients. Intensive Care Med..

[6] Kim M.H., Ahn J.Y., Song J.E., Choi H., Ann H.W., Kim J.K., Kim J.H., Jeon Y.D., Kim S.B., Jeong S.J. (2015). The C-reactive protein/albumin ratio as an independent predictor of mortality in patients with severe sepsis or septic shock treated with early goal-directed therapy. PLoS ONE.

[7] Ranzani O.T., Zampieri F.G., Forte D.N., Azevedo L.C., Park M. (2013). C-reactive protein/albumin ratio predicts 90-day mortality of septic patients. PLoS ONE.

[8] Siekmann W., Eintrei C., Magnuson A., Sjolander A., Matthiessen P., Myrelid P., Gupta A. (2017). Surgical and not analgesic technique affects postoperative inflammation following colorectal cancer surgery: A prospective, randomized study. Colorectal. Dis..

[9] Arima K., Yamashita Y.I., Hashimoto D., Nakagawa S., Umezaki N., Yamao T., Tsukamoto M., Kitano Y., Yamamura K., Miyata T. (2017). Clinical usefulness of postoperative C-reactive protein/albumin ratio in pancreatic ductal adenocarcinoma. Am. J. Surg..

[10] Sun F., Ge X., Liu Z., Du S., Ai S., Guan W. (2017). Postoperative C-reactive protein/albumin ratio as a novel predictor for short-term complications following gastrectomy of gastric cancer. World J. Surg. Oncol..

[11] Li N., Tian G.W., Wang Y., Zhang H., Wang Z.H., Li G. (2017). Prognostic role of the pretreatment C-reactive protein/albumin ratio in solid cancers: A meta-analysis. Sci. Rep..

[12] Joen J.S., Ji S.M. (2015). Diagnostic value of procalcitonin and CRP in critically ill patients admitted with suspected sepsis. J. Dent. Anesth. Pain Med..

[13] Jing X., Huang C., Zhou H., Li C., Fan L., Chen J., Zhang G., Liu Y., Cui Z., Qi D. (2015). Association between serum C-reactive protein value and prognosis of patients with non-small cell lung cancer: A meta-analysis. Int. J. Clin. Exp. Med..

[14] Munteanu A., Munteanu D., Tigan S., Bartos A., Iancu C. (2017). How do surgical stress and low perioperative serum protein and albumin impact upon short term morbidity and mortality in gastric cancer surgery?. Clujul. Med..

[15] Sonoda A., Ohnishi S., Nakao S., Iwashita Y., Hashimoto N., Ishida K., Kondo Y., Ishitsuka Y., Irie T. (2015). Factors affecting serum albumin in the perioperative period of colorectal surgery: A retrospective study. BMC Res. Notes.

[16] Shiota G., Umeki K., Okano J., Kawasaki H. (1995). Hepatocyte growth factor and acute phase proteins in patients with chronic liver diseases. J. Med..

[17] Nakamura N., Hatano E., Iguchi K., Seo S., Taura K., Uemoto S. (2016). Posthepatectomy liver failure affects long-term function after resection for hepatocellular carcinoma. World J. Surg..

